# A Constant-Force Technique to Measure Corneal Biomechanical Changes after Collagen Cross-Linking

**DOI:** 10.1371/journal.pone.0105095

**Published:** 2014-08-27

**Authors:** Olivier Richoz, Sabine Kling, Souska Zandi, Arthur Hammer, Eberhard Spoerl, Farhad Hafezi

**Affiliations:** 1 Dept. of Ophthalmology, Geneva University Hospitals, Geneva, Switzerland; 2 Dept. of Ophthalmology, University Hospital Dresden, Dresden, Germany; 3 Dept. of Ophthalmology, Keck School of Medicine, University of Southern California, Los Angeles, California, United States of America; Cardiff University, United Kingdom

## Abstract

**Purpose:**

To introduce a constant-force technique for the analysis of corneal biomechanical changes induced after collagen cross-linking (CXL) that is better adapted to the natural loading in the eye than previous methods.

**Methods:**

For the biomechanical testing, a total of 50 freshly enucleated eyes were obtained and subdivided in groups of 5 eyes each. A Zwicki-Line Testing Machine was used to analyze the strain of 11 mm long and 5 mm wide porcine corneal strips, with and without CXL. Before material testing, the corneal tissues were pre-stressed with 0.02 N until force stabilization. Standard strip extensiometry was performed as control technique. For the constant-force technique, tissue elongation (Δ strain, %) was analyzed for 180 seconds while different constant forces (0.25 N, 0.5 N, 1 N, 5 N) were applied.

**Results:**

Using a constant force of 0.5 N, we observed a significant difference in Δstrain between 0.26±0.01% in controls and 0.12±0.03% in the CXL-treated group (p = 0.003) over baseline. Similarly, using a constant force of 1 N, Δstrain was 0.31±0.03% in controls and 0.19±0.02% after CXL treatment (p = 0.008). No significant differences were observed between CXL-treated groups and controls with 0.25 N or 5 N constant forces. Standard stress-strain extensiometry failed to show significant differences between CXL-treated groups and controls at all percentages of strains tested.

**Conclusion:**

We propose a constant-force technique to measure corneal biomechanics in a more physiologic way. When compared to standard stress-strain extensiometry, the constant-force technique provides less variability and thus reaches significant results with a lower sample number.

## Introduction

Corneal biomechanics are of increasing importance in understanding the pathophysiology of corneal weakening, thinning, and irregularity in diseases such as keratoconus and postoperative ectasia. They also determine the outcome after refractive laser surgery. Corneal stiffness can be modified by corneal collagen cross-linking (CXL), a procedure that uses UV-A light and riboflavin to increase the mechanical and biochemical stability of corneal stroma [1]–[5]. CXL is effective in delaying or arresting the progression of keratoconus [1], [6]–[10] and postoperative corneal ectasia [11]–[13] as demonstrated *in vitro and in vivo* when the limitations of the technique are respected [14], [15].

Only recently CXL was transformed from a laboratory technique to a widely used clinical method [1], [3], [4], [16]. As with all new technologies, continuous modifications and improvements of the original protocol are tested both experimentally [17]–[21] and clinically [13], [22], [23]. Initially, CXL has relied on *in vitro* biomechanical studies with stress-strain measurements that were adapted from standard material testing, using values in a non-physiological range that do not accurately measure biomechanical changes that are relevant in the physiological and pathological biological tissue like the cornea [1], [4], [24].

The standard biomechanical measurement method (stress-strain extensiometry) analyzes the force necessary to induce a progressive strain in the corneal tissue [3], [4] and serves to characterize the elastic material properties. It was adapted from methods for the mechanical analysis of metals and polymers with rather homogeneous chemical or molecular bonds. In contrast, the mechanical properties of a biological tissue depend on chemical bonds and molecular interactions that may be distributed inhomogeneously and lead to time-dependent, i.e. viscoelastic material properties. Also, the process of cross-linking introduces a variety of new chemical bonds [25], having an effect on the microstructural interactions. These modifications are difficult to measure accurately using conventional stress-strain methods.

Another limitation of standard biomechanical testing is that it is not tension-constant. In the natural environment corneas are hardly subjected to a steady increasing force (such as it is applied in stress-strain tests), it rather has to withstand the normal and constant intraocular pressure (IOP). In this study we introduce a method using a constant-force, which will allow us to analyze the temporal and hence viscoelastic biomechanical properties of the cornea and their changes with CXL.

## Methods

### Corneal collagen cross-linking (CXL)

50 fresh enucleated porcine eyes were obtained from a local abattoir (Abattoirs d'Orbe SA), stored at 5°C and prepared for the experiments within less than 6 hours after harvest. Only corneas with an intact epithelium, lack of focal stromal edema and a pachymetric thickness of 800±100 µm, as measured by ultrasound pachymetry (SP-2000, Tomey Corporation, Nagoya, Japan), were used. Debridement of the corneal epithelium was performed with a hockey blade. A solution containing 0.9% NaCl (no preservative agent) and 0.1% riboflavin (vitamin B2) was instilled onto the cornea every 2 minutes for 25 minutes. Corneas were then exposed to UV-A irradiation (CXL-365, Peschke Meditrade, Cham, Switzerland) with a large diameter aperture (11 mm), at 9 mW/cm^2^ for 10 minutes at a working distance of 5 cm. After 5 minutes of irradiation, riboflavin drops were instilled once to minimize changes in corneal hydration. Controls were deepithelialized and received riboflavin instillation, but were not exposed to UV-A irradiation.

### Biomechanical measurements

After CXL or control preparation, one corneo-scleral strip (5 mm width, full thickness) was obtained centrally in the horizontal axis from each eye. 4 mm of the end of each strip were dedicated to fixation, leaving no more than 11 mm of central corneal strip length, so that the entire central strip had been irradiated and cross-linked. For tensile strength measurement, we used a Zwicki-Line Testing Machine (Zwick, Ulm, Germany), calibrated with a distance accuracy of 2 µm and a tensile sensor with no more than 0.21% of measurement uncertainty between 0.25 N and 50 N. The Zwick Z 0.5 is a classical extensiometer composed of a linear holder extension arm whose speed can be controlled and a Newton meter, which measure the real time Force in Newton exerted by the arm on the held specimen. The conversion from force to stress is calculated from the thickness and width of the specimen. Corneal strips were fixed using pneumatics grips with 164 N. Data analysis was performed using the Xpert II-Testing Software for Static Testing Systems (Zwick, Ulm, Germany).

Before starting either conventional strip-extensiometry tests or constant-force measurements, the corneal tissue was pre-stressed with 0.02 N until force stabilization was achieved. This pre-stressing is equivalent to an IOP of about 6 mmHg.

**Figure 1:**
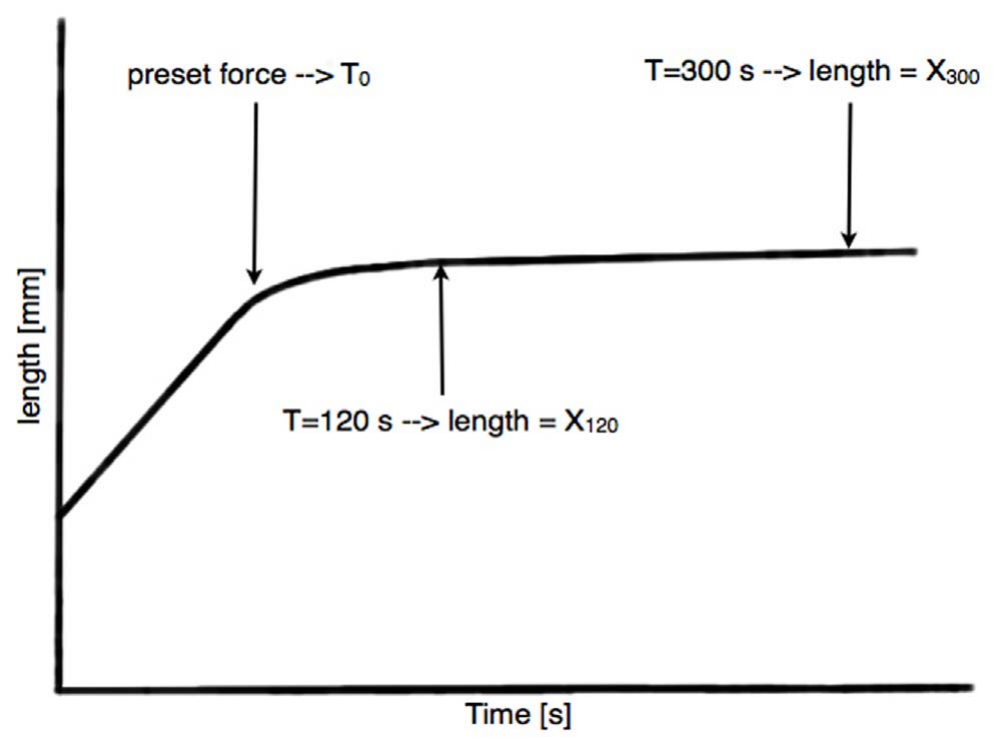
Schematic illustration of the constant-force method. T_0_ was determined when the preset force was reached. At t = 120 s and t = 300 s, respectively, the distances X_120_ and X_300_ were measured. Comparisons were made between the induced strain during the time period from 120 s to 300 s.

For the new constant-force measurements (n control = 20 eyes, n CXL = 20 eyes), force was measured every 39 milliseconds. Strain was applied at a speed of 1 mm/min up to a constant force of 0.25 N, 0.5 N, 1 N, and 5 N (n = 5 eyes per force and group). These forces are equivalent to 58, 115, 231 and 1154 mmHg, respectively. Time started (T_0_) when the pre-set force was reached. Then the amount of absolute strain was measured at 120 s and 300 s, corresponding to X_120_ and X_300_, respectively (Figure 1) and Δstrain was determined within this time period (being 0% at 120 s).

Conventional strip-extensiometry (n(control) = 5 eyes, n(cxl) = 5 eyes) was then performed at a speed of 1 mm/min where force was recorded as a function of strain. The testXpert II software was used to calculate the Young's moduli (elasticity moduli) at 2, 4, 6, 8, and 10% of strain and to analyze the variance.

For both methods, the total testing time was less than 6 minutes per strip. During and after each test, all junctions between the pneumatic grips and the corneal strips were checked and if any movement or rupture between the strips and grips was suspected, the test was not used for the subsequent analyses.

### Statistical Analysis

Statistical analysis was performed using *testXpert* II software (Zwick Roell Group, Ulm, Germany). Values were expressed as average ± standard deviation (SD). Differences between the experimental groups were evaluated by the students t-test and considered statistically significant (*) or highly significant (**), when the probability value (*P*) was <0.05 or <0.01, respectively.

## Results

**Figure 2:**
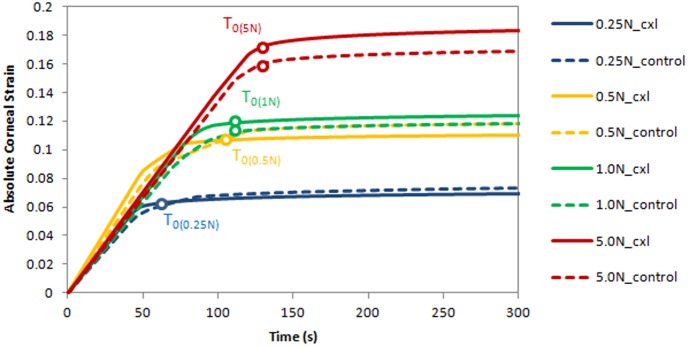
Absolute corneal strain as a function of time under different constant forces. Dashed lines represent control corneas, continuous lines cross-linked corneas. The higher the applied force, the later it was reached (T_0_) and the larger strains were observed.

Figure 2 depicts the elongation of cross-linked and control corneal strips over time under various applied forces (0.25 N, 0.5 N, 1 N, 5 N) using the constant-force technique. The corneal strip elongated (slope of the curve) until the point of stabilization (horizontal portion of the curve). After the pre-determined force was reached, elongation stabilized within less than 150 seconds. The time to reach stabilization as well as the amount of strain correlated positively with the applied force: stabilization (R^2^ = 0.79) occurred more rapidly and strains (R^2^ = 0.87) were lower in tests using a lesser force.

**Figure 3:**
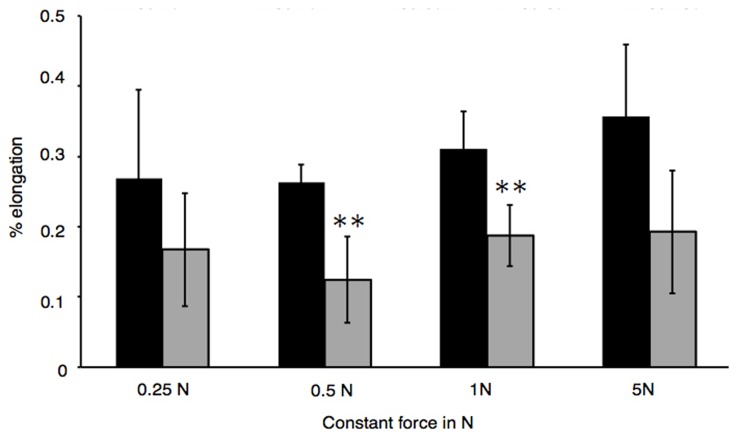
Biomechanical properties of corneal strips and significance from constant-force technique. T_0_ was fixed at the beginning of the elongation, corresponding to X_120_ (t = 120). ΔStrain under constant forces of 0.25, 0.5, 1 and 5 N (n = 5 eyes per force) was determined after 180 seconds, corresponding to X_300_ (t = 300). *P<0.05; **P<0.01.

Using the constant-force technique, significant differences between CXL and control groups were observed (Figure 3), when a constant force of 0.5 N or 1 N was applied. Under a constant force of 0.5 N, untreated corneas elongated by 0.26±0.01% while CXL-treated corneas increased in length by 0.12±0.03% (p = 0.003). When 1 N of constant force was applied, CXL-treated corneas elongated by 0.19±0.02% and non-irradiated controls by 0.31±0.03% (p = 0.008). No significant differences were observed between CXL-treated groups and controls with 0.25 N or 5 N of applied force.

**Figure 4:**
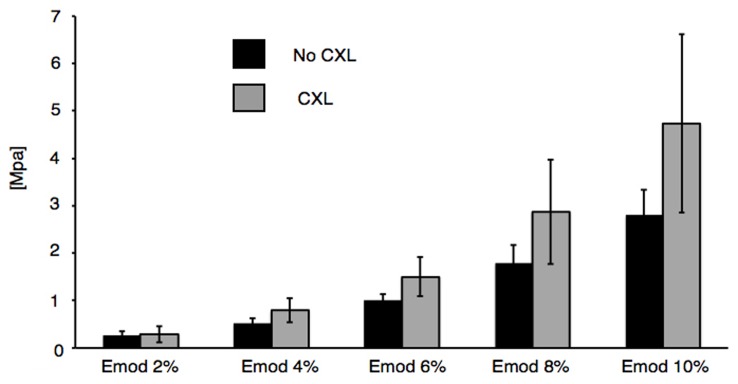
Biomechanical properties of corneal strips and significance from conventional testing. Young's modulus/elasticity modulus (emod %) at 2, 4, 6, 8, and 10% of strain in cross-linked corneas and untreated controls (MPa, n = 5 eyes for both, cxl and control).

Figure 4, shows the different elasticity modulus of corneal strips with and without CXL treatment using the conventional stress-strain method. The absolute values for all elasticity moduli in the CXL-treated group were higher than in non-irradiated controls, but did not reach significance: The elasticity modulus at 6% strain was 1.5±0.21 MPa in the CXL group and 1.01±0.06 MPa in non-irradiated controls (p = 0.06). At 2% of strain, the elasticity modulus was nearly indistinguishable between the two groups, with 0.29±0.09 MPa for the CXL group and 0.26±0.05 MPa for the non-irradiated controls (p = 0.78).

The measurement repeatability was similar in both techniques −0.0017 in the constant-force technique and 0.103 MPa in standard stress-strain extensiometry – which corresponds to a relative standard deviation of about 10%.

## Discussion

CXL is currently widely used to strengthen the cornea in keratoconus, pellucid marginal degeneration and ectasia after refractive laser surgery. Systems (Ocular Response Analyzer, Corvis ST) based on analyzing corneal deformation following an air-puff have been used to evaluate the effect of CXL in vivo [10], [26]–[28]. A major disadvantage of these techniques is that the recorded geometrical deformation parameters are strongly dependent on the IOP and corneal thickness and do not represent a real mechanical tissue properties. Further factors that impede an accurate measurement of in vivo corneal biomechanics is the relatively high inter-individual variability due to age [29], smoking habits [30]–[32], and the hormonal status of estrogens [15], [33], [34] and thyroid hormones [35], [36]. Ex vivo techniques are therefore still preferred when corneal stiffness needs to be quantified.

Since the surface/volume ratio of a corneal strip is high, rapid dehydration prior to testing was a concern [37]. We therefore performed all biomechanical tests in less than 6 minutes, minimizing tissue dehydration due to evaporation. To transfer the current constant-force technique to human cadaver eyes, forces will have to be reduced by approximately a third in order to account for the difference in thickness between the average porcine (800 µm) and human cornea (550 µm) [20], [38].

The corneal stroma is mainly composed of collagen fibers and proteoglycans [39]. Recent studies suggest that the corneal stiffening after CXL seems to be due to the creation of additional covalent bonds between collagen fibers and proteoglycans [40], [41].

Stress-strain extensiometry in corneas has been derived from elastic material testing, where increasing loads are applied, typically until material break. In the clinical setting however, corneal biomechanics are especially interesting under constant loading conditions. Here, we propose a method that uses constant forces (0.25 N to 5 N) to test biomechanical properties, which might better reflect the physiological loading condition due to the IOP. The applied forces were chosen in a similar range than in previous stress-strain extensiometry, covering IOPs from 58 to 1154 mmHg. Using the constant force method significant changes after cross-linking were found in two of four forces, while standard stress-strain extensiometry could not find any difference. Therefore our results suggest that the constant force approach is probably more accurate and reliable in detecting differences after CXL than standard stress-strain testing as it addresses the viscoelastic material properties instead of purely elastic properties. Thereby the sensitivity of the constant force technique is force dependent: At low forces, creep behavior is small and depends mainly on the extracellular matrix. With increasing force, the load is more and more carried by the collagen fibers. As significant differences were only found for intermediate forces, we may conclude that cross-linking affects the interaction between collagen fibers and the extracellular matrix and not one of them alone.
